# Ligand Functionality-Dependent Performance of Organotin Carboxylate Resists

**DOI:** 10.3390/mi17010001

**Published:** 2025-12-19

**Authors:** Xiaofei Liu, Tianren Liu, Kaixin Su, Jingxin Lei, Yuanfu Chen, Yuan Chen, Dongxu Yang

**Affiliations:** 1 State Key Laboratory of Optical Field Manipulation Science and Technology, Institute of Optics and Electronics, Chinese Academy of Sciences, Chengdu 610209, China; 2 School of Integrated Circuit Science and Engineering, University of Electronic Science and Technology of China, Chengdu 611731, China; 3 State Key Laboratory of Advanced Polymer Materials, Polymer Research Institute, Sichuan University, Chengdu 610065, China

**Keywords:** metal-containing resist, deep-ultraviolet lithography, organotin carboxylate, radical-mediated reaction, pattern transfer

## Abstract

As metal-containing resists attract increasing research interest in high-resolution lithography, gaining insights into the photochemical mechanisms, particularly in relation to the ligand functionality, is actively demanded. In this work, a controlled pair of organotin carboxylates with analogous structures but different functional groups has been designed and synthesized as deep-ultraviolet (DUV) resists. Both resists demonstrate 90 nm half-pitch resolution and the capability of pattern transfer on carbon-based hard-mask layers. Through various characterizations and comparison of the controlled pair, we propose two competitive reaction paths for the organotin system with olefin groups, which regulate the lithographic sensitivity and dissolution contrast. Our findings highlight the structural adjustability of organotin carboxylates and their potential application as high-resolution and etch-durable DUV resists.

## 1. Introduction

For over three decades, semiconductor industry has been relying on chemically amplified resists (CARs) as the major patterning material for advanced lithography [1,2,3,4]. First introduced in KrF lithography, CAR adopts a combination of acid-labile matrix polymer and photo-acid generator (PAG) to realize a photo-initiated, acid-catalyzed deprotection process, which perfectly circumvents the conflict of resist transparency and light utilization efficiency [5,6,7]. Based on this mechanism, a series of high-contrast polymers have been developed, including poly-hydroxystyrene platform for KrF lithography, poly-methacrylate platform for ArF lithography, etc. Although CAR has been successfully extended to extreme ultraviolet (EUV) lithography, it starts to show limitations when the feature size approaches 10 nm and below. The main factor lies in the diffusive nature of the catalytic process, rendering a balanced relationship among resolution, line edge roughness, and sensitivity, known as the RLS trade-off [8,9,10]. In addition, organic compounds generally have weak absorption within the EUV band, which magnifies the statistical effect and shot noise during latent image formation [11,12]. Recently, significant attention has been drawn to new material platforms based on metal-containing resists, owing to their high EUV absorption, small and uniform molecular size, non-diffusive mechanism, and excellent etch resistance [1,13,14,15,16,17]. Specifically, tin-based metal oxide resists have exhibited sub-10 nm resolution capability, demonstrating significant potential in next-generation high-NA and hyper-NA EUV lithography [18,19,20]. Regardless of EUV absorption, the advantages of metal-containing resists are also applicable in high-resolution deep-ultraviolet (DUV) lithography in advanced nodes due to their exceptionally high etching resistance, outstanding pattern fidelity, and superior resistance to pattern collapse. Several metal-containing materials have been reported to exhibit lithographic performance in DUV exposure. For example, Yeh et al. reported a cobalt-doped zinc methacrylate as a photosensitive precursor, which showed negative-tone behavior under 193 nm radiation with good pattern quality [21]. Other research groups, including Nandi et al. and Ober et al., also used DUV exposure to characterize and analyze the radiation mechanism for various metal-containing electron-beam or EUV resists [21,22,23,24].

Metal-containing resists with a wide variety of molecular structures have been reported. For instance, metal oxide nanoparticle resists typically contain an inorganic core and organic ligands, forming a uniform core–shell structure with good solubility in organic solvents [24,25,26,27,28,29]. Depending on the type of additives, the possible exposure mechanism has been proposed to be ligand exchange and nanoparticle condensation [30,31,32,33]. Metal-oxo cluster resists take up another important category with a more compact molecular structure and improved lithographic performance compared to the nanoparticle resists. Similarly, the proposed exposure mechanism involves the photolyzation of organic ligands, followed by the agglomeration of activated clusters [34,35,36,37]. In addition to nanoparticles and nanoclusters, another type of metal-containing resists consists of a complex configuration with metal ions and ligand groups, named organometallic resists. With a more “organic” molecular structure, organometallic resists show better compatibility with standard solvents and developers [38]. However, the exposure mechanism of organometallic resists can be diverse, depending on the ligand functionality [1,36]. Overall, the detailed photochemical process remains unclear for the majority of metal-containing resists due to the participation of both metal center and organic ligands, which may provide multiple reaction paths and complex products.

To establish a deeper understanding of the role of ligand functionality in metal-containing resists, organotin carboxylates were employed in this work due to their high synthetic flexibility, which allows for the preparation of well-defined molecular compounds via straightforward routes and enables precise structural tuning. We herein design and synthesize a controlled pair of organotin carboxylates for DUV lithography: Bn_2_Sn(MAA)_2_, featuring reactive double-bond functionality; and Bn_2_Sn(*i-*BA)_2_, with a highly similar structure but lacking double bonds. The photochemical mechanism, particularly in relation to the influence of olefin groups, has been investigated through a series of spectroscopic characterizations. Furthermore, the resolution capability and corresponding pattern quality of the organotin carboxylate resists, as well as their potential for pattern transfer in a bilayer configuration, have been assessed.

## 2. Materials and Methods

### 2.1. Materials

Benzyl chloride (BnCl), tin powder (Sn), propylene glycol methyl ether acetate (PGMEA), dichloromethane (DCM), and isopropanol (IPA) were purchased from Aladdin. Sodium isobutyrate, sodium methacrylate, and anisole were purchased from Macklin, and Kermel, respectively. Organic bottom anti-reflective coating (BARC) was obtained from PhiChem Corporation (Shanghai, China). Spin-on carbon (SOC) was purchased from HengKun New Materials Technology Co., Ltd. (Xiamen, China). All chemical reagents were used as received without further purification.

### 2.2. Measurements

Fourier transform infrared (FTIR) spectra were obtained with a Nicolet iS50 spectrophotometer (Thermo Fisher; Waltham, MA, USA). Nuclear magnetic resonance (NMR) spectra were recorded on a Bruker Avance 600 MHz spectrometer (Billerica, MA, USA). The film thicknesses were measured using spectroscopic ellipsometer (SENTECH SE 850 DUV, Berlin, Germany). Thermogravimetric (TG) analysis was performed using Mettler TGA/DSC 3+ (Zurich, Switzerland). AFM (Atomic Force Microscope) were measured using a Bruker Multimode 8 ( Bruker; Billerica, MA, USA). The scanning electron microscopy (SEM) images were obtained by a Hitachi SU8000 (Tokyo, Japan). The line edge roughness (LER) was measured using SUMMIT software (V10.9.3). The etching process was performed using Sentech/Etchlab 200. Post-exposure solubility was evaluated based on the residual film thickness remaining after blanket DUV exposure (500 mJ/cm^2^) and a 30 s development process. X-ray photoelectron spectroscopy (XPS) analysis was performed using a Thermo Scientific ESCALab 250Xi spectrometer (Thermo Fisher; Waltham, MA, USA). Raman spectroscopy was collected using a Renishaw inVia Qontor confocal Raman microscope (Wotton-under-Edge, UK).

### 2.3. Synthesis of Bn_2_Sn(MAA)_2_ and Bn_2_Sn(i-BA)_2_

Tin powder (17.8 g, 0.15 mol) was thoroughly mixed with water (3 mL) to form a paste. This paste was then dispersed in toluene (150 mL) under intense agitation and brought to 140 °C. Benzyl chloride (19.2 g, 0.15 mol) was introduced drop by drop over a period of 3 min, and the mixture was maintained under reflux for 3 h. The resulting mixture was subjected to hot filtration. The filtrate was then cooled to afford metallic luster crystals upon subsequent filtration. These crystals were dissolved in toluene at 120 °C, followed by another hot filtration. This entire recrystallization procedure was repeated three times, ultimately yielding metallic, silky needle-like crystals. ^1^H NMR (Bn_2_SnCl_2_): 7–7.2 (m, 10H), 3.1 (m, 4H).

In a separate procedure, a 50 mL round-bottom flask equipped with a magnetic stirrer was charged with sodium carboxylate (RCOONa, 4 mmol) and dichloromethane (10 mL). Triethylamine (0.56 g, 4 mmol) was added gradually to the stirred solution. After stirring for 5 min, a solution of dibenzyltin dichloride (0.5 g, 2 mmol) in dichloromethane (10 mL) was introduced into the reaction vessel. The reaction was allowed to proceed for 3 h until completion. The mixture was subsequently filtered and the filtrate was concentrated under reduced pressure to obtain the amorphous target product [39]. ^1^H NMR (Bn_2_Sn(MAA)_2_): 7.0–7.2 (m, 10H), 6.0 (s, 2H), 5.5 (s, 2H), 3.0 (m, 4H), 1.8 (s, 6H); Bn_2_Sn(*i*-BA)_2_: 7.0–7.3 (m, 10H), 3.0 (m, 4H), 2.4 (m, 2H), 1.0 (m, 12H).

### 2.4. Preparation of Photoresist Solutions

The photoresist solutions were prepared by mixing Bn_2_Sn(MAA)_2_/Bn_2_Sn(*i*-BA)_2_ and PGMEA solvent and filtered through a 0.22 μm polytetrafluoroethylene filter membrane. The photoresist films were prepared by spin-coating photoresist solutions onto silicon wafers at a rotation speed of 2500 rpm and subsequently dried in hot plate.

### 2.5. Lithography Procedures

On the surface of silicon wafers pre-coated with SOC material (thickness ~80 nm), the photoresist solution was spin-coated at 2500 rpm for 30 s. Subsequently, a soft bake was performed on a precision hot plate at 80 °C for 5 min to remove residual solvents. The dried photoresist film (thickness ~70 nm) was subjected to DUV interference lithography, followed by a post-exposure bake (PEB) at 100 °C for 60 s. The DUV interference lithography (DUV-IL) system was built based on our previous work [40,41]. Development was completed in IPA (developing for 20 s and rinsing for 5 s), ultimately forming periodic line/spatial patterns.

### 2.6. Etching Procedures

The grating patterns were transferred into the SOC layer via an oxygen plasma etch using a gas mixture of O_2_ (10 sccm) and Ar (3 sccm), carried out at an RF power of 50 W and a chamber pressure close to the base pressure, which yielded an etch rate of approximately 15 nm/min. This was followed by etching into the underlying silicon substrate using a fluorine-based plasma composed of CHF_3_ (12 sccm), SF_6_ (4 sccm), and Ar (3 sccm) at an RF power of 100 W and a chamber pressure close to the base pressure, resulting in an etch rate of about 28 nm/min. The final etch depth reached approximately 100 nm.

### 2.7. Sensitivity Curve

Different regions on the same wafer coated with photoresist films were exposed separately by adjusting the exposure time to achieve exposure effects with different doses (*D*). The thickness of the residual photoresist in each exposed region was measured three times using a spectroscopic ellipsometer (three sets of data were collected for each region), and the average value was calculated. Finally, the normalized residual thickness (*T*/*T*_max_, where *T*_max_ is the maximum measured value) was curve-fitted with the exposure dose (*D*) to obtain the sensitivity curve [38].

## 3. Results

**Figure 1:**
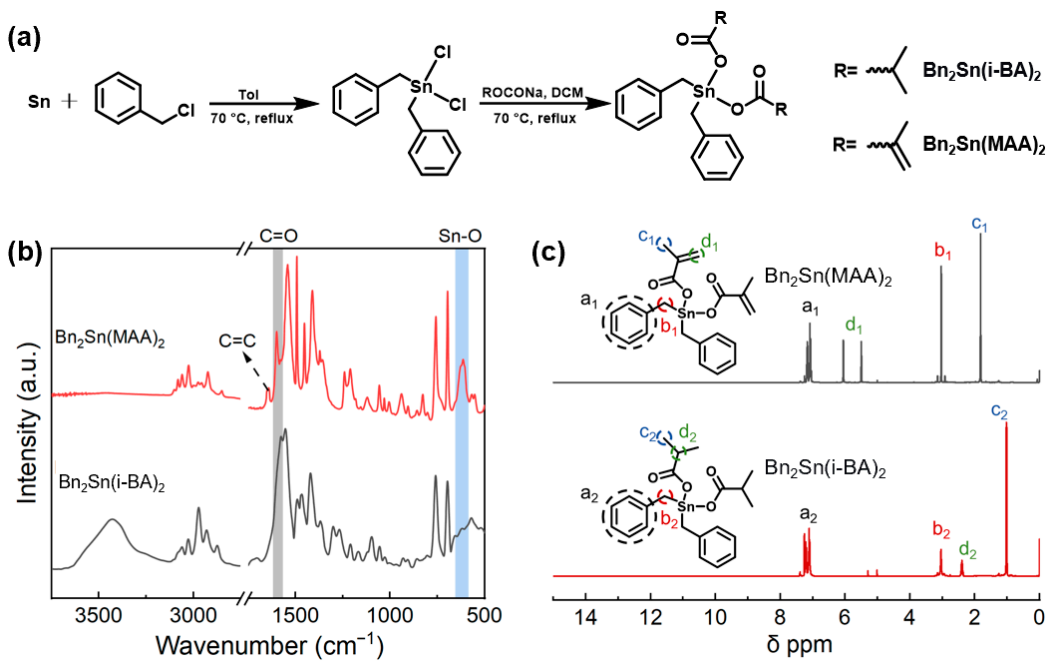
(**a**) Synthetic method, (**b**) Fourier transform infrared (FTIR) spectra, and (**c**) ^1^H NMR spectra of Bn_2_Sn(MAA)_2_ and Bn_2_Sn(*i-*BA)_2_.

In order to investigate the role of ligand functionality in metal-containing resists, we designed and synthesized two highly analogous organotin complexes with simple and clear structures, named Bn_2_Sn(MAA)_2_ and Bn_2_Sn(*i-*BA)_2_. The synthetic method is displayed in Figure 1a. While both shared a common intermediate of dibenzyl tin dichloride, Bn_2_Sn(MAA)_2_ and Bn_2_Sn(*i-*BA)_2_ were obtained by substituting the chlorides with methacrylic acid (MAA) and isobutyric acid (*i-*BA), respectively. Differing only in a specific bonding motif (C=C vs. C-C) on the ligands, these two molecules were designed as a controlled pair for comparative studies. The synthesized materials were characterized by FTIR and ^1^H NMR spectroscopies. The FTIR spectra of both Bn_2_Sn(MAA)_2_ and Bn_2_Sn(*i-*BA)_2_ showed signals at 613 cm^−1^ and 1599 cm^−1^, which can be assigned to the characteristic absorption peaks of Sn-O and C=O, respectively (Figure 1b). As expected, the spectrum of Bn_2_Sn(MAA)_2_ shows an extra C=C stretching vibration peak at 1640 cm^−1^. Detailed structural information was further revealed via ^1^H NMR characterization. In Bn_2_Sn(MAA)_2_ sample, the peaks of vinyl protons (δ 6.0 and δ 5.5 ppm), aromatic protons (δ 7.0 ppm), methylene protons (δ 3.0 ppm), and methyl protons (δ 1.8 ppm) can be clearly identified. Similarly, the location of characteristic peaks in Bn_2_Sn(*i-*BA)_2_ sample also matched well with the target structure. Overall, FTIR and ^1^H NMR spectroscopies confirmed the successful synthesis of the two organotin carboxylates.

**Figure 2:**
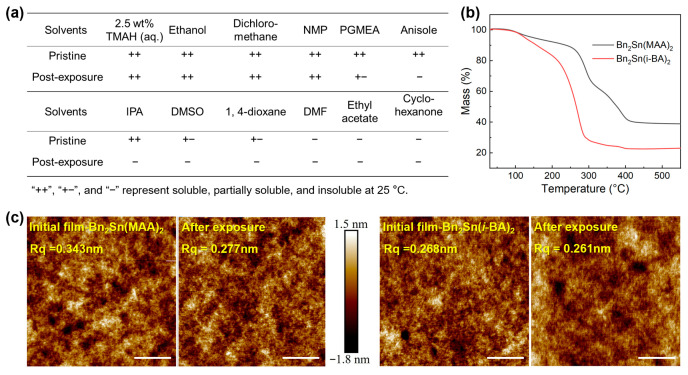
(**a**) The solubility of Bn_2_Sn(MAA)_2_ and Bn_2_Sn(*i-*BA)_2_ before and after exposure. Note the two resists show similar solubility behavior. (**b**) Thermogravimetric (TG) curves of Bn_2_Sn(MAA)_2_ and Bn_2_Sn(*i-*BA)_2_. (**c**) Atomic force microscope (AFM) images of Bn_2_Sn(MAA)_2_ and Bn_2_Sn(*i-*BA)_2_ before and after exposure (scale bar: 500 nm).

The physicochemical properties critical to lithographic performance were evaluated, including solubility, thermal stability, and film quality. A solubility screening was first performed using common organic solvents, as shown in Figure 2a. The dissolution property of Bn_2_Sn(MAA)_2_ and Bn_2_Sn(*i*-BA)_2_ was found to be identical in the tested solvents, possibly due to their structural similarity. The pristine materials exhibited favorable solubility in ethanol, dichloromethane, propylene glycol methyl ether acetate (PGMEA), isopropyl alcohol (IPA), and anisole. Therefore, PGMEA was chosen as the casting solvent due to its compatibility in lithographic processes. Subsequently, the post-exposure solubility was tested on spin-coated films with a flood exposure. As shown in Figure 2a, IPA (selected as developer) and anisole exhibited the largest dissolution contrast before and after exposure. To determine the baking conditions of the resist film, the thermal stability of Bn_2_Sn(MAA)_2_ and Bn_2_Sn(*i-*BA)_2_ was evaluated via TG analysis (under nitrogen atmosphere). While both resists showed an onset of ligand decomposition at ~100 °C, Bn_2_Sn(MAA)_2_ exhibited a slower mass loss and a larger residual mass till the final stage (Figure 2b). Based on the structural difference between the two materials, such behavior was likely due to the thermal polymerization of the olefins in Bn_2_Sn(MAA)_2_, which rendered the decomposed products less volatile and partially retained as carbonaceous residuals. In contrast, the majority of organic moieties in Bn_2_Sn(*i*-BA)_2_ sample were cleared, leaving tin-oxide species as the primary residue [42]. Based on the above analysis, a relatively low-temperature and long-duration pre-bake condition of 80 °C for 5 min was set to ensure removal of residue solvents and prevent thermal degradation. Smooth spin-casted films with surface roughness below 0.4 nm (root-mean-square value) were obtained under this condition, which further reduced to sub-0.3 nm after flood exposure (Figure 2c). The results suggest that both Bn_2_Sn(MAA)_2_ and Bn_2_Sn(*i*-BA)_2_ formed a high-quality amorphous film, which persisted during exposure without crystallization.

**Figure 3:**
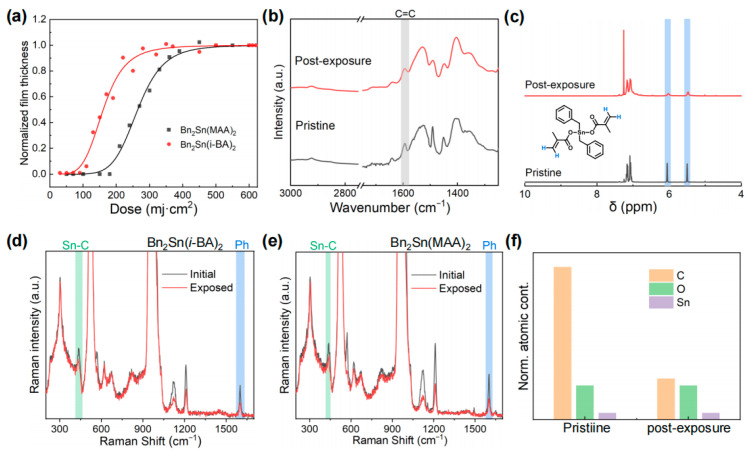
(**a**) Sensitivity curves of Bn_2_Sn(MAA)_2_ and Bn_2_Sn(*i-*BA)_2_. (**b**) FTIR spectra, and (**c**) ^1^H NMR spectra of Bn_2_Sn(MAA)_2_ before and after DUV exposure. (**d**) Raman spectrum of Bn_2_Sn(*i*-BA)_2_ before and after exposure. (**e**) Raman spectra of Bn_2_Sn(MAA)_2_ before and after exposure. (**f**) Normalized atomic contents of Bn_2_Sn(MAA)_2_ extracted from XPS. Carbon and oxygen contents were normalized by tin element.

The lithographic performance of Bn_2_Sn(MAA)_2_ and Bn_2_Sn(*i-*BA)_2_ was evaluated using IPA as developer. As displayed in Figure 3a, the DUV (266 nm) response curves of the two resists show a negative-tone behavior, with the sensitivity of Bn_2_Sn(*i-*BA)_2_ (160 mJ/cm^2^) significantly higher than that of Bn_2_Sn(MAA)_2_ (262 mJ/cm^2^). Interestingly, a similar contrast of ~3 was obtained for both resists, based on the calculation described in the Experimental Section. To investigate the role of the olefin group in the photochemical process, the resist films of Bn_2_Sn(MAA)_2_ before and after exposure (receiving a high dosage of 500 mJ/cm^2^) were characterized through FTIR and NMR spectroscopies, focusing on the signal of the carbon double bond. While FTIR spectra exhibit a slight weakening of the characteristic C=C peak at 1640 cm^−1^ after exposure, NMR shows a clear reduction in olefinic proton peaks with chemical shift between 5.5 and 6.0 ppm, indicating considerable consumption of double bond upon irradiation (Figure 3b,c). Raman spectroscopy was performed to monitor the ligand decomposition after exposure (Figure 3d,e). The intensity decrease in the peaks at ~1600 cm^−1^ (assigned to the benzene ring) and ~438 cm^−1^ (assigned to Sn-C bonds) after DUV exposure indicates the cleavage of benzyl Sn-C bonds and formation of volatile species that can escape from the resist film. Furthermore, the elementary change during exposure was characterized. Assuming that Sn atoms remained constant (i.e., no volatile tin compound generated during exposure), the carbon and oxygen contents were normalized by tin element. As shown in Figure 3f, a significant loss of carbon was observed after exposure, which is consistent with the results from Raman spectroscopy.

**Figure 4:**
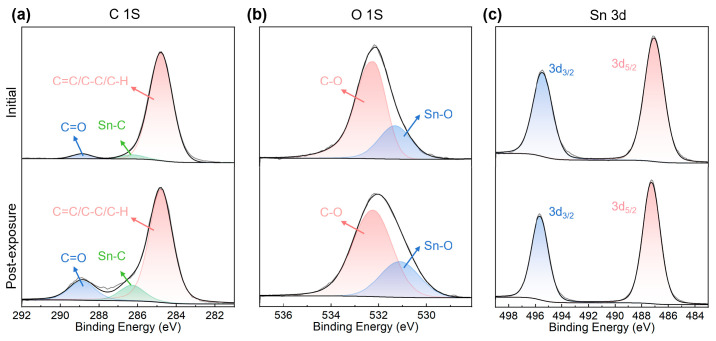
XPS of Bn_2_Sn(MAA)_2_ pristine film, and DUV exposed film: (**a**) C 1s spectra; (**b**) O 1s spectra; (**c**) Sn 3d spectra.

The chemical state of Bn_2_Sn(MAA)_2_ was analyzed by XPS. As shown in Figure 4a, the C 1s spectra exhibited identical peak positions before and after exposure, indicating no significant change in the chemical environment. The relative increase in C=O and Sn-C bonds after exposure may result from the loss of benzyl groups, as mentioned above. In the O 1s spectra (Figure 4b), both the as-deposited and post-exposure samples can be fitted with two characteristic peaks, corresponding to Sn-O bonds (~531.3 eV) and C-O bonds (~532.3 eV). After exposure, the relative proportion of Sn-O bonds increased, suggesting partial loss of carboxylate ligands and possible formation of condensed tin-oxide species. Furthermore, the Sn 3d spectra (Figure 4c) revealed a slight upward shift in binding energy upon exposure, reflecting a subtle change in the local chemical environment after the ligand transition.

**Scheme 1:**
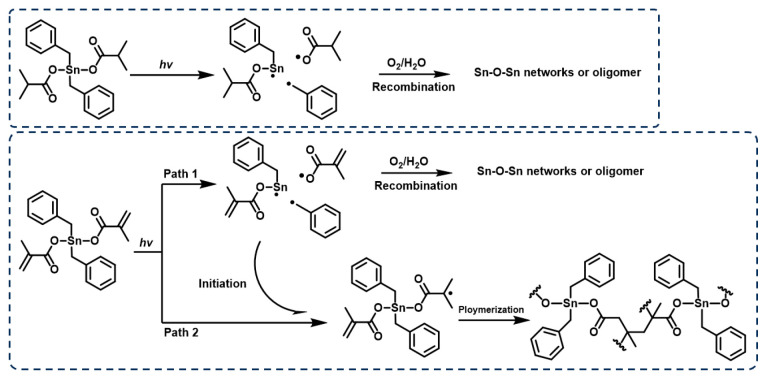
Proposed exposure mechanism diagram of Bn_2_Sn(MAA)_2_ and Bn_2_Sn(*i-*BA)_2_.

Based on the reported mechanism study, high-energy radiation to organometallic resists induces a homolytic cleavage of organic ligands and formation of free radicals. The radicals then recombine or initiate a condensation process in the presence of H_2_O/CO_2_/O_2_ to form metal-oxo networks or oligomers, which applies to the Bn_2_Sn(*i-*BA)_2_ sample in this study (Scheme 1) [3,43,44,45]. In the case of Bn_2_Sn(MAA)_2_, a competitive reaction path is proposed due to the reactivity of free radicals with olefin groups, triggering crosslinking of the metal complex molecules (Scheme 1). Compared with metal-oxo network or oligomer formation, such olefin crosslinking process is supposed to have lower dissolution conversion efficiency due to a lack of polarity change. Therefore, the coexistence of the two competitive reaction paths renders Bn_2_Sn(MAA)_2_ less sensitive compared to its olefin-free counterpart, which is consistent with the chemical characterization and lithographic evaluation. Furthermore, competitive DUV absorption by the double bonds in Bn_2_Sn(MAA)_2_ may also contribute to the deterioration of sensitivity.

**Figure 5:**
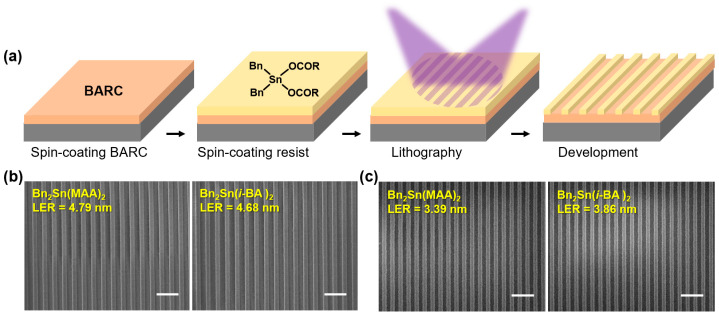
(**a**) The schematic diagram of the DUV interference lithography process. SEM images of the interference fringes at (**b**) 110 nm half-pitch and (**c**) 90 nm half-pitch for Bn_2_Sn(MAA)_2_ and Bn_2_Sn(*i-*BA)_2_ (scale bar: 500 nm).

The resolution capability of Bn_2_Sn(MAA)_2_ and Bn_2_Sn(*i-*BA)_2_ was investigated using a home-built DUV interference lithography setup with a dual-beam configuration, in which the grating pitch size is adjustable (Figure 5a). Interference fringes with half-pitches of 110 nm and 90 nm were used to assess resolution and pattern quality, with the exposure dose optimized to achieve a 1:1 line/space ratio. To reduce the standing-wave effect, a commercial bottom anti-reflective coating (BARC) for KrF lithography was applied beneath the resist layer. As shown in Figure 5b,c, both Bn_2_Sn(MAA)_2_ and Bn_2_Sn(*i-*BA)_2_ clearly resolved the gratings down to 90 nm with sharp pattern edges and sub-5 nm line edge roughness (LER), indicating smooth and near-vertical resist sidewalls. The remarkable resolution and pattern quality of organotin carboxylates can be attributed to their high dissolution contrast (Figure 3a). Notably, the sub-100 nm resolution enters the range of ArF dry lithography, demonstrating the potential of this material family as a high-performance DUV resist platform.

**Figure 6:**
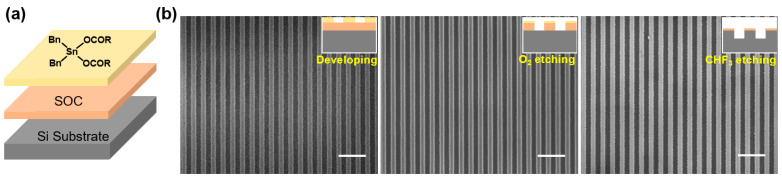
(**a**) Schematic diagram of a bilayer film structure with Bn_2_Sn(MAA)_2_ resist and spin-on-carbon layer. (**b**) SEM image of 90 nm half-pitch resist pattern and transferred patterns into SOC layer and into silicon substrate (scale bar: 500 nm).

Due to the additional inorganic composition, metal-containing resists generally possess significantly higher etch resistance compared to the organic resists [46,47]. Particularly, the enhanced etch durability in O_2_ plasma enables these resists to serve as hard masks for amorphous carbon or spin-on-carbon (SOC) layers, thereby simplifying the traditional tri-layer process flow to a bilayer process. Motivated by this, we replaced the organic BARC layer with an 80 nm-thick SOC layer (Figure 6a), which also has sufficient absorption to eliminate the bottom reflection. Using Bn_2_Sn(MAA)_2_ as the resist, well-defined 90 nm half-pitch gratings were patterned without collapse or peeling, indicating good adhesion at the resist-SOC interface (Figure 6b). The gratings were subsequently transferred into SOC (by O_2_ plasma) and silicon substrate (by fluorine-based plasma) with a final transfer depth of ~100 nm. As shown in Figure 6b, the high fidelity of the etched pattern demonstrates the potential of organotin carboxylate resist in nano-feature transfer.

## 4. Conclusions

In summary, we have presented a pair of structurally tailored organotin carboxylates as high-resolution photoresists for deep-ultraviolet lithography. One of the resists contains a reactive olefinic functionality on the ligand, and the other has a highly analogous structure but lacking double bond. While a general radical-mediated condensation process is assumed, we found that the presence of additional olefin groups may introduce a new competitive reaction path of ligand crosslinking. Due to the lower extent of polarity conversion, the addition of this reaction path led to a sensitivity modulation. However, both resists demonstrated sub-100 nm resolution with high pattern quality. In addition, the high etch resistance under O_2_ plasma enables these resists transferrable on carbon-based hard-mask layers. The presented results in this study highlight the understanding of ligand functionality in organotin resists, demonstrating their potential in advanced deep-ultraviolet lithography. Due to the non-chemically amplified nature of organotin carboxylates, their sensitivity remains lower than that of commercial DUV resists. We propose that the sensitivity of this resist family can be further optimized through ligand engineering to reduce the dissociation energy, or through introduction of photosensitizers/co-initiators to provide additional reactive species. Relative work will be carried out in our follow-up studies.

## Data Availability

The original contributions presented in this study are included in the article. Further inquiries can be directed to the corresponding author.
